# Editorial: Occupational risks of healthcare personnel

**DOI:** 10.3389/fpubh.2022.1022327

**Published:** 2022-09-21

**Authors:** Helena C. Maltezou, Begoña Martínez-Jarreta, Venerando Rapisarda, Caterina Ledda

**Affiliations:** ^1^Directorate of Research, Studies, and Documentation, National Public Health Organization, Athens, Greece; ^2^Forensic and Occupational Medicine, Department of Pathological Anatomy, Forensic and Legal Medicine and Toxicology, University of Zaragoza, Zaragoza, Spain; ^3^Occupational Medicine, Department of Clinical and Experimental Medicine, University of Catania, Catania, Italy

**Keywords:** healthcare personnel, healthcare workers, occupational, infection, vaccination, psychological stress, vaccine, COVID-19

Healthcare personnel (HCP), and especially those working in front-line roles, are at increased risk for occupational exposure to a variety of infections, including vaccine-preventable diseases (1). At the same time, HCP are exposed not only to environmental risks (e.g., biological, physical, and chemical risks) but also to psychological stress, which was exceptionally intensified during the coronavirus disease 2019 (COVID-19) pandemic. As such, ensuring safety within healthcare facilities and creating health-promoting workplaces is becoming increasingly relevant in a globalizing workplace (2).

It was an honor for us and a challenge at the same time to accept an invitation by *Frontiers in Public Health* and serve as Topic Editors for the Research Topic: “*Occupational risks of healthcare personnel*.” The aim of this Research Topic was to provide an overview of occupational risks of exposure and illness among HCP in the context of the COVID-19 pandemic, covering a wide range of occupational risks, from vaccine-preventable diseases to psychological issues and violence which has increased dramatically the past years.

Trevisan et al. studied a cohort of 11,022 students of a medical school in Italy and found excellent vaccination compliance, high seropositivity, and high antibody titers against rubella. This study highlights the importance of high vaccination coverage rates and immunity levels among healthcare students before joining the healthcare workforce. Yet, vaccine hesitancy has been recognized by the World Health Organization as a top threat to global health (3). Despite the deployment of mRNA COVID-19 vaccines < 1 year after the declaration of the COVID-19 pandemic, vaccine hesitancy emerged as a major public health obstacle for the global efforts to control the pandemic and speed the return to normality. Particularly for HCP, COVID-19 vaccination significantly reduces their COVID-19-associated morbidity but also episodes and duration of absenteeism during periods of excess healthcare demand (4). A cross-sectional study conducted in May 2021 by Iguacel et al. showed high levels of negative attitudes toward vaccines in up to 22.6% of participants, yet only 1.5% of them refused to get the COVID-19 vaccine. Notably, HCP did not show higher rates of positive attitudes toward vaccines compared with non-HCP while they showed higher rates of vaccination refusal, which is of concern. In addition, Gopalakrishnan et al. reported gaps in infection control training for HCP and fear to provide healthcare to COVID-19 patients in hospitals in India, despite the fact that most HCP had adequate knowledge about COVID-19 and practiced safety precautions adequately, which should be addressed.

The present Research Topic has also covered many topics related to the mental health of HCP during the COVID-19 pandemic. Resilience is a major topic: Afshari et al., Goff et al., and Gillie et al. identified psychosocial and demographic predictive factors that may contribute to greater resilience among HCP during the COVID-19 pandemic. The findings of the abovementioned studies can be used to implement psychosocial interventions, in particular for front-line HCP.

Another issue is violence in the healthcare workplace. Pina et al. and Ma et al. investigated on the identification of sources of conflict and causes of violence in hospital and indicate that frontline HCP urgently need relevant parties to take effective measures in terms of legislation, security, and dispute handling capacity, to prevent the occurrence of violence and protect medical personnel's safety.

Emphasis was also placed on the impact of burnout on HCP. Song et al., Xiao et al., and Ahmed et al. analyzed the role of burnout as a mediating factor between the three types of emotional labor strategies and presenteeism among HCP. The results suggested that interventions should be enhanced in vulnerable groups to reduce burnout and promote wellbeing.

Mental health disorders were studied by Salgado de Snyder et al., Wang et al., and Riedel et al. These research groups analyzed occupational and personal stressors, mental health indicators, perceived discrimination, and help-seeking behaviors among HCP.

In addition, Yuan et al. investigated the characteristics of neck-shoulder pain (NSP) among HCP and highlighted that concern from supervisors about workers' health, and the ability of workers to change their shift status to off duty when they were not feeling well was shown to induce significant effects to NSP. This shows that effective employee involvement can mitigate risks in the workplace. A similar approach carried out by Secosan et al. studied the frontline HCP's positive psychological state—PsyCap—impacts on anxiety/depression and burnout/mental health complaints. PsyCap is a crucial variable that may decrease the impact of anxiety and depression on psychological outcomes such as emotional exhaustion, inefficacy, and psychological problems among Romanian medical professionals working on the frontline during the COVID-19 pandemic. Thus, psychological interventions that help medical staff gain personal resources are appropriate in the context of the COVID-19 pandemic.

Stress among HCP during the COVID-19 pandemic was investigated by Ali et al. and Sun et al. These studies recommend that constructive planning and necessary provision of supportive measures by the legal authorities and policymakers protect nurses and minimize their psychological stress to fulfill high-quality nursing care. Sleep disorders were studied by Li et al. The latter investigation associated shift work-sleep disorder with scheduling strategies and personal behavior during the COVID-19 pandemic. Santoro et al. contributed to evidence the dermatological effects among HCP during the COVID-19 pandemic and indicated the development of prevention strategies in the workplace in order to improve the wellbeing of HCP and reduce the impact of dermatological adverse reactions to personal protective equipment. Wang and Yang investigated the influence of professional identity on E-Learning adaptability among Chinese nursing students during COVID-19. Lastly, Isonne et al. highlighted several challenging areas and critical issues relating to working conditions. Job satisfaction plays an important role in healthcare organization and management; it is critical for maintaining and improving staff efficiency and consequently the quality of care provided.

## Conclusions

It is indisputable that appropriate health and safety management in the healthcare sector is essential to providing quality care to patients; likewise, HCP are exposed to a wide range of risks in their workplaces that require adequate preventive measures.

However, the COVID-19 pandemic has highlighted, as never before, the weaknesses and shortcomings of occupational risk prevention in the healthcare sector. Adding to that, it has made visible key issues such as the vaccination of HCP, the mandatory vaccination policies, the psychosocial risks derived from the health emergency (stress caused by work pressure, the risk of contagion, and the lack of means to protect their health and that of others, the fact of facing an unknown agent or suffering aggressive behavior from patients, etc.). All of them have become main concerns during the pandemic, as it is clearly reflected in this Research Topic, and are amenable to deep reflection.

The COVID-19 pandemic has shown that adequate protection of HCP from exposure to risks at work is still suboptimal and their improvement must be considered a priority also in the event of a sanitary crisis.

This Research Topic brings together works that contribute to increasing the scientific evidence on the occupational risks of HCP and promote cogitation for the present and future challenges in this field, including preparedness to face new emergencies and preventive measures to implement.

## Author contributions

All authors listed have made a substantial, direct, and intellectual contribution to the work and approved it for publication.

## Conflict of interest

The authors declare that the research was conducted in the absence of any commercial or financial relationships that could be construed as a potential conflict of interest.

## Publisher's note

All claims expressed in this article are solely those of the authors and do not necessarily represent those of their affiliated organizations, or those of the publisher, the editors and the reviewers. Any product that may be evaluated in this article, or claim that may be made by its manufacturer, is not guaranteed or endorsed by the publisher.
